# Control of Self-Winding Microrobot Using an Electromagnetic Drive System: Integration of Movable Electromagnetic Coil and Permanent Magnet

**DOI:** 10.3390/mi15040438

**Published:** 2024-03-25

**Authors:** Hao Li, Zhaopeng Zhang, Xin Yi, Shanhai Jin, Yuan Chen

**Affiliations:** 1 Department of Mechatronics and Information Engineering, Shandong University at Weihai, Weihai 264209, China; yustlh@sdu.edu.cn; 2 Department of Mechanical Engineering, Yanbian University, Yanji 133002, China; rocman215@outlook.com (Z.Z.); yxin0912@gmail.com (X.Y.)

**Keywords:** microrobot, electromagnetic drive system, synchronized control, permanent magnet

## Abstract

Achieving precise control over the motion position and attitude direction of magnetic microrobots remains a challenging task in the realm of microrobotics. To address this challenge, our research team has successfully implemented synchronized control of a microrobot’s motion position and attitude direction through the integration of electromagnetic coils and permanent magnets. The whole drive system consists of two components. Firstly, a stepper motor propels the delta structure, altering the position of the end-mounted permanent magnet to induce microrobot movement. Secondly, a programmable DC power supply regulates the current strength in the electromagnetic coil, thereby manipulating the magnetic field direction at the end and influencing the permanent magnet’s attitude, guiding the microrobot in attitude adjustments. The microrobot used for performance testing in this study was fabricated by blending E-dent400 photosensitive resin and NdFeB particles, employing a Single-Layer 4D Printing System Using Focused Light. To address the microrobot drive system’s capabilities, experiments were conducted in a two-dimensional and three-dimensional track, simulating the morphology of human liver veins. The microrobot exhibited an average speed of 1.3 mm/s (movement error ± 0.5 mm). Experimental results validated the drive system’s ability to achieve more precise control over the microrobot’s movement position and attitude rotation. The outcomes of this study offer valuable insights for future electromagnetic drive designs and the application of microrobots in the medical field.

## 1. Introduction

With the evolution of medical technology, medical robots are increasingly employed in various precision surgeries, including cerebral neurosurgery, plastic surgery, cardiac repair, and artificial joint replacement [1]. They also continually forge new applications in assisted medicine [2]. One such emerging field is in vivo micro-medical robots, representing a novel dimension in medical surgical robots. These robots possess the capability to minimize trauma, navigate within human body cavities, utilize human body cavities and liquid mediums for locomotion, and meticulously perform tasks such as diagnosis, drug delivery, and surgery within the body. These characteristics contribute significantly to the advancement of modern medical technology. Magnetic micro/nano robots designed for biomedical applications offer crucial insights for achieving effective targeted therapy, showcasing substantial prospects for future applications.

To propel and manage diminutive robots, various electromagnetic actuators have been employed, utilizing diverse control techniques for wirelessly propelling microrobots [3]. These minuscule, wirelessly operated robots can access restricted spaces, including sensitive internal bodily sites, offering precise and reproducible approaches for minimally invasive interventions and targeted diagnostics and therapeutics at the cellular level. This encompasses the provision of secure and dependable medical treatments for targeted therapies.

In the magnetic control method, current drive methods are primarily categorized into two types [4]: Firstly, employing the magnetic field generated by multiple pairs of fixed electromagnetic coils allows precise control of the magnetic field strength and direction within the microrobot’s motion area by regulating the current’s size and direction inside the coils [5]. However, generating a sufficiently strong magnetic field and achieving an extensive movement control range demands larger currents and coil sizes for the fixed electromagnetic coils. As early as 2010, Michael P. Kummer et al., from ETH Zurich achieved five degrees of freedom in microrobot motion using eight orthogonal electromagnetic coils, featuring a single coil with 712 turns and a drive current of 20 A [6]. While the experiment successfully achieved motion control of the microrobot, it nonetheless exhibited several noteworthy limitations. The power supply boasts a substantial amount of power, yet its operational range is quite limited. Furthermore, its ability to produce a magnetic field of only 15 mT hinders its practicality for long-distance applications. Gilgueng Hwang and his colleagues from the Laboratoire Photonique et Nanostructures in France utilized a smaller four-coil electromagnetic drive system to propel a bilayer microrobot composed of PMN-PT and nickel. This electromagnetic coil, consisting of 630 turns, carried a current of 1 A. However, this setup confined the positioning control of the microrobot to a small area measuring 4 mm in length and 3 mm in width [7]. Additionally, the fixed position and limited number of coils restricted the microrobot’s movement to a two-dimensional space. Meanwhile, Kim et al., from the Korea Institute of Medical Microrobotics proposed a redundant control method deploying ten electromagnetic coils to facilitate microrobot motion within a field, achieving free motion in a stomach model and a live pig. The experiment involved a maximum of 968 turns of coils and a drive current of 20 A [8]. Gwangjun Go et al., from Johns Hopkins University, Baltimore, Maryland, USA, accomplished motion control of a microrobot with two rotational degrees of freedom and three translational degrees of freedom using nine electromagnetic coils, where the coils used for the experiments comprised 1368 turns [9]. The microrobot was capable of maneuvering within a designated area of 120 mm in diameter. Nevertheless, given the intricate design of the electromagnetic coils, there is a pressing need to significantly expand the coils if the intention is to carry out future experiments involving organisms or even human subjects. This undertaking presents a formidable challenge pertaining to coil architecture, power distribution, and numerous other considerations. Additionally, Yun Kim et al., from Hanbat National University, Daejeon, South Korea, demonstrated motion control of a cluster of microrobots using a field-free point system consisting of eight electromagnetic coils with 828 turns, generating a magnetic field strength of 20 mT [10]. Chan Kim and his team at Chonnam National University in Korea employed six electromagnetic coils to create free points, enabling targeted manipulation of ferromagnetic fluids. These coils boasted a maximum of 671 turns, generating a peak magnetic field gradient of 1.5 T/m. Nevertheless, the system’s effectiveness was limited to approximately 10 mm, posing difficulties in experiments requiring long-distance control [11]. Kim Tien Nguyen and his colleagues at the Korean Institute of Medical Microrobots, leveraging particle imaging technology, successfully tracked magnetic microparticles within living mice using free-point driving systems with varying coil configurations: four, six, and nine coils. These experiments utilized electromagnetic coils powered by a maximum current of 10 A, drawn from a 5000 W power source [12].

An alternative driving approach involves utilizing a mechanical structure to control the movement of a microrobot by driving a permanent magnet. Permanent magnets offer advantages over electromagnetic coils, including high magnetic field strength (up to 1 T or more), strong traction force, simple driving structure, a wide control range, and lower driving cost. Nevertheless, the connection between the permanent magnet and the mechanical structure limits its degree of freedom to that of the mechanical structure, resulting in less flexible motion posture control compared to an electromagnetic coil. The two-dimensional cargo platform at Southern Methodist University utilizes a mechanical structure to drive permanent magnets, thereby controlling the positioning and movement of a population of alginate-based microrobots. The two-dimensional cargo platform at Southern Methodist University utilizes a mechanical structure to drive permanent magnets, thereby controlling the positioning and movement of a population of alginate-based microrobots. However, due to the lack of rotational capabilities and high-level control precision, this mechanism can only achieve two-dimensional movement of the microrobots, preventing it from extending into three-dimensional space and realizing attitude control of the microrobots [13]. Xinjian Fan and colleagues at the School of Mechanical Engineering, Suzhou University, achieved control over the movement and posture of magnetic fluid by integrating a permanent magnet with an electromagnetic coil [14]. The driving distance was 25 cm, and the power supply for the electromagnetic coil was only 25 V. The electromagnetic drive system developed in the study was limited to two-dimensional motion, precluding its utilization in three-dimensional space. Chuang Li and the team from the Department of Biomedical Engineering, University of Groningen, accomplished the spiral forward movement of the microrobot by propelling a permanent magnet to rotate and move using a motor [15]. Nevertheless, the study’s posture control of the microrobot was limited due to the application of the rotating effect of the external magnetic field in their spiral rotation. Chong Hong et al., from the Max-Planck-Institute for Intelligent Systems, Germany, manipulated a micro-gear group with a Halbach array, generating a torque of up to 0.182 mNm at a speed of 40 Hz [16]. However, due to the use of a fixed Habach array in the study, steering of the microrobot was not achievable.

**Figure 1:**
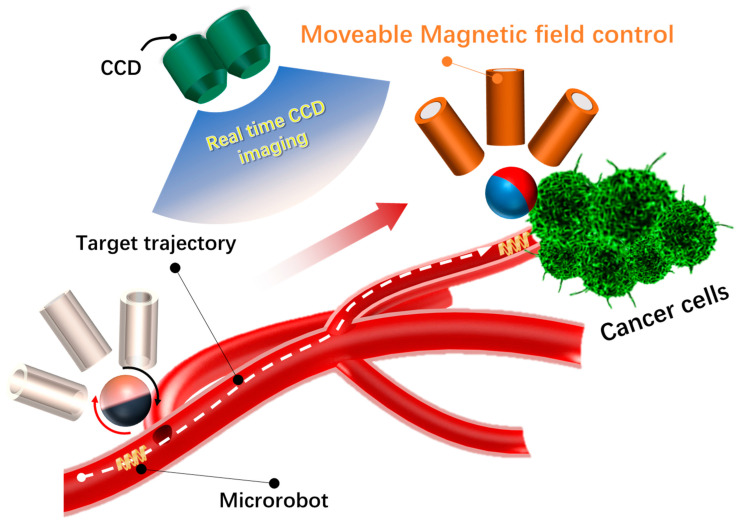
Illustrates the schematic diagram depicting the electromagnetic drive concept for microrobot.

Building on prior studies, this paper introduces a novel magnetic manipulation system that utilizes moving coils and a magnetic sphere to govern the motion and attitude of a microrobot within a track simulating a blood vessel model. This design enhances the precision of the drive system controlling the microrobot’s motion position and attitude rotation, depicted in Figure 1. Initially, we fabricated a spiral-shaped magnetically driven microrobot using a Single-Layer 4D Printing System Using Focused Light. Subsequently, we positioned it within a tailored track.

Using a delta-driven structure, multiple coils are strategically positioned close to the starting point of the designated track [17,18,19]. These coils generate the required magnetic field to align the microrobot for motion. Concurrently, electromagnetic coils dynamically control the orientation of the NdFeB magnetic sphere in real time, enhancing the magnetic field strength and indirectly influencing the motion and attitude of the magnetic microrobot, as depicted in Figure 1. The whole novel magnetic manipulation system is designed to be lightweight, comprising three stepper motors, three electromagnetic coils, and a NdFeB magnetic sphere. Notably, the electromagnetic coil employed in this study comprises only 350 turns, a size smaller than those commonly utilized in similar studies. Moreover, the ratio between the effective workspace volume of the entire drive system and the volume of the delta structure is more flexible compared to existing systems.

The electromagnetic drive magnetic manipulation system is designed to manipulate the microrobot, enabling it to reach the specified position within a designated spatial area ensuring precise movement. Additionally, during the movement process, the system allows for adjusting the attitude of the microrobot based on the specific direction of movement. This capability enhances its adaptability to the movement environment, especially in narrow areas.

## 2. Materials and Methods

### 2.1. Magnetic Microrobot

**Figure 2:**
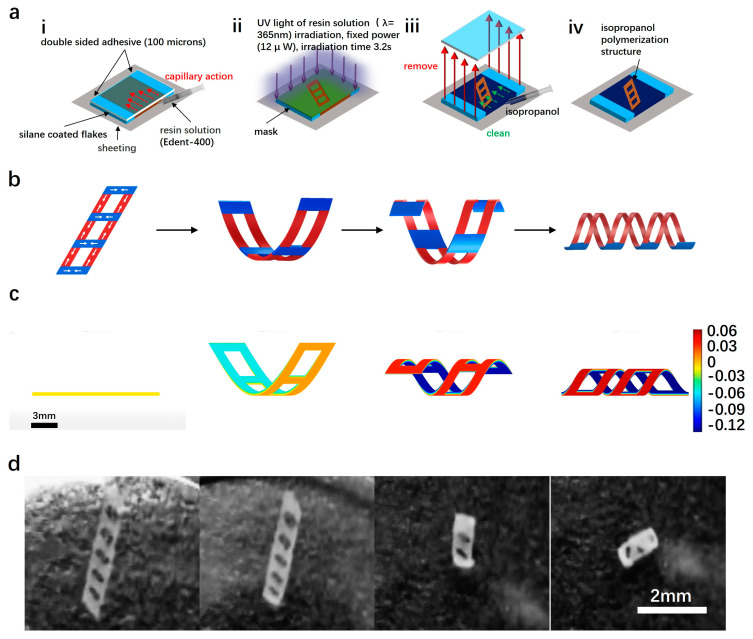
Flowchart of the process of microrobot fabrication. (**a**) Preparation process of the microrobot. (i) Two transparent slides, each with areas of 24 × 24 mm and 20 × 20 mm, were prepared. Double-sided tape (thickness: 100 μm; Nitto, Inc.) was then applied between them to create the microchamber. (ii) Expose a mixed resin solution to focused UV light (λ = 365 nm) at a fixed power (12 µW) for 3.2 s. (iii) After a 2 h soak in isopropyl alcohol (IPA), peel off the coverslips and retain the cured pattern structure. (iv) Obtain the UV-cured microrobot precursor structure. (**b**) Microrobot deformation mechanism, where the reagent mixture continues to harden under UV illumination, increasing the degree of curing as the harder portion increases. (**c**) Strain simulation analysis of the cured structure. (**d**) Actual deformation process of the patterned structure rolled according to the initial design (scale bar: 2 mm).

In this study, we adopt the authors’ previous research results: conventional 4D printing of microrobots. Briefly, the requirement for two layers with distinct physical properties, such as Young’s modulus, coefficient of thermal expansion, density, and pH, leads to constraints associated with material selection and extended printing times [20,21,22,23]. Utilizing focused light printing technology (SPROUT), microrobots are fabricated using E-dent400 photosensitive resin. The longitudinal density gradient of this monolayer material corresponds to the photon density gradient, creating a microrobot designed for electromagnetic actuation. The fabrication process is depicted in Figure 2a:

(i) Two transparent slides, each with areas of 24 × 24 mm and 20 × 20 mm, were prepared. Double-sided tape (thickness: 100 μm; Nitto, Inc., Osaka, Japan) was then applied between them to create the microchamber.
(ii) For the biocompatible resin solution, a mixture of 50 wt% of e-dent 400 resin and 50 wt% NdFeB particles was prepared using a plenary mixer at 2000 rpm for 30 min.
(iii) The resin solution was injected into the chamber via capillary force using a syringe. The microchamber was placed in the sample holder and cured with UV light (λ = 365 nm) for 6 s.
(iv) The exposed sample was immersed in a Petri dish filled with isopropyl alcohol (IPA) and covered with aluminum foil for 2 h. Subsequently, the slide glass and tape were removed and rinsed with additional IPA to remove any un-polymerized ink. The patterned membranes adhered to the cover glass. Finally, after several minutes, the patterned membranes self-detached and rolled according to the initial design. (Figure 2d).

In the presence of focused light, the photon gradient received by the E-dent400 photosensitive resin increases continuously with depth. Consequently, the lower layer of the resin develops a greater longitudinal density gradient, resulting in a smaller and harder structure compared to the upper layer. As the light exposure time increases, the photosensitive resin continues to cure, leading to and increased thickness of the harder layer and gradual enhancement in the degree of curvature of the polymeric structure. Figure 2c illustrates the strip-like numerical structure exhibiting an augmented curling effect with prolonged light exposure, eventually forming a spiral structure.

While determining the optimal curing level for achieving the best curling effect is crucial during the curing and deformation of resin structures, it is notable that microrobots produced by 4D printing often involve the combination of different materials to induce shape changes. Therefore, the deformation simulation for micro-structures in this study employs two materials with distinct properties, simplifying the resin structure in the simulation process. The deformation of the polymerized structure is shown in Figure 2c, using a numerical simulation method. After conducting the simulation analysis and evaluating the fabrication effect, it is observed that the optimal ratio of Young’s modulus between the soft and hard layers of the polymerized structure is 2:8, with an optimal thickness ratio of 8:2. The resin structure achieves the maximum curling effect when maintaining this ratio between the soft and hard layers of the polymerized structure [24,25,26].

### 2.2. Electromagnetic Drive System

#### 2.2.1. Electromagnetic Drive System Design

**Figure 3:**
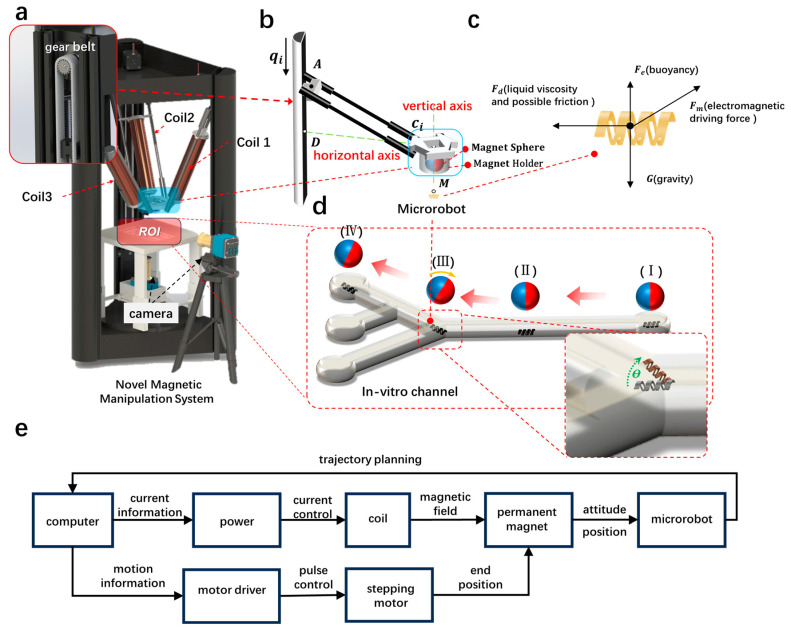
Electromagnetic drive system analysis. (**a**) Schematic diagram of the electromagnetic drive system. (**b**) Kinematic analysis of the electromagnetic drive system. (**c**) Force analysis of the microrobot. (**d**) Schematic diagram of the microrobot being pulled by the electromagnetic drive system in the experimental track. (**e**) Flowchart illustrating the relationship between the modules of the electromagnetic drive system.

The movable range of the permanent magnet in the drive system (Figure 3a) constitutes a cylindrical space with a diameter of 27 cm and a height of 66 cm. The coil sleeve, crafted from pure aluminum, includes a coil wound with 350 turns of double-layer copper wire, each with a 1 mm diameter. Below the coil, a freely rotatable NdFeB magnetic sphere with a 30 mm diameter presents a surface magnetic induction strength of 5000 Gs. The orientation of the sphere is manipulated by the magnetic field generated by the electromagnetic coil, governing the microrobot’s attitude. Power is supplied to the electromagnetic coil by the ITECH IT6942A (Hengkaikeji, Inc., Luoyang, China) programmable DC power supply. The LabVIEW control program oversees the experimental power supply, determining the output current for each power supply based on the direction of the end movement of the delta structure. This adjustment alters the magnetic field direction at the end and controls the orientation of the magnetic sphere.

The drive system is controlled through a NET_AMC3XER V1.1 three-axis motion control card, three C-DR42A stepper motor drivers, and three fulsun42 stepper motors as the power source. The stepper motors feature a 1.8° stepping angle, a torque of 420 mN·m, and a maximum running rate of 2000 PPS. The LabVIEW program manages the operation of the stepper motors. Motion coordinate points at the end of the structure are input through the front panel of the LabVIEW control program, which then transmits the number and direction of motion pulses for each motor to NET_AMC3XER V1.1. It is responsible for generating corresponding pulses to the stepper motor drivers to determine the direction and number of steps. The entire motion experiment is recorded and observed using two HDC60 cameras, f-4–12 mm, 1600 wpixel, MOKOSE (Yunlangtechnologyco, Ltd., Shenzhen, China)—one positioned beneath the experimental platform and the other at the side of the platform.

#### 2.2.2. The Kinematic Analysis of Electromagnetic Drive Systems

The kinematic analysis of one of the coils within the delta structure during the stepper motor drive process is depicted in Figure 3b. In this representation, M signifies the position of the magnetic microrobot; B marks the middle point of the end structure situated directly above point M; C_i_ represents the midpoint of the end structure connected with the connecting rod; point D denotes the support rod at an equal height with both point B and point C_i_; A designates the endpoint of the sliding position; q_i_ indicates the motion displacement of the connecting slider, also representing the distance between the connecting slider and the stepper motor; and a_i_ (i = 1, 2, 3) specifies the angle of the connecting rod for every two sets of adjacent connecting rods. The length of the connecting rod ∣AC_i_∣ = l, and the height of the whole support rod is h.

In the illustration, point A is defined by the coordinates (x_A_, y_A_, z_A_), point M by (x_M_, y_M_, z_M_), and point B by (x_M_, y_M_, z_B_) due to its location just above point M. Concerning the end structure, assume its intermediate point B is at a distance of I_BC_ from the connecting midpoint C_i_. This allows us to derive the coordinates of point C_i_ using the following formula [27]:

(1) $$C_{i}=\left[\begin{array}{c} x_{C}\\ y_{C}\\ z_{C} \end{array}\right]=\left[\begin{array}{c} x_{B}+l_{BC}\cos\alpha_{i}\\ y_{B}+l_{BC}\sin\alpha_{i}\\ z_{B} \end{array}\right]=\left[\begin{array}{c} x_{M}+l_{BC}\cos\alpha_{i}\\ y_{M}+l_{BC}\sin\alpha_{i}\\ z_{B} \end{array}\right],\left[\alpha_{i}=\frac{2\pi }{3}\cdot \left(i-1\right)\right],$$

Since point D is on the same support bar as point A and is located at the same height as point B, the coordinates of point D are (x_A_, y_A_, z_B_). It can be obtained by association with Equation (1):

(2) $$\left|C_{i}D\right|=\sqrt{{\left(x_{A}-x_{B}-l_{BC}\cos\alpha_{i}\right)}^{2}+{\left(y_{A}-y_{B}-l_{BC}\sin\alpha_{i}\right)}^{2}},$$

The distance between point A and point D is obtained from ∣C_i_ D∣ and the length l of the connecting rod:

(3) $$\left|AD\right|=\sqrt{l^{2}{-\left(x_{A}-x_{B}-l_{BC}\cos\alpha_{i}\right)}^{2}-{\left(y_{A}-y_{B}-l_{BC}\sin\alpha_{i}\right)}^{2}},$$

This yields q_i_:

(4) $$q_{i}=h-z_{B}-\sqrt{l^{2}{-\left(x_{A}-x_{B}-l_{BC}\cos\alpha_{i}\right)}^{2}-{\left(y_{A}-y_{B}-l_{BC}\sin\alpha_{i}\right)}^{2}},$$

According to Equation (4), the distance and direction of motion of each stepper motor can be obtained from the coordinate values of the microrobot at the position of the nodes of the motion trajectory (M_1_, M_2_, M_3_, M_4_...):

#### 2.2.3. Control of Magnetic Sphere Direction in the Electromagnetic Drive System

This study proposes the utilization of multiple electromagnetic coils to generate an electromagnetic field, enabling control over the attitude direction of the magnetic sphere. Subsequently, the microrobot’s attitude is manipulated through the alteration of the magnetic field through the magnetic sphere’s direction, as illustrated in Figure 3d. The magnetic field generated by a single electromagnetic coil at any point in space $\vec{B_{e}}$ can be calculated by the Biot–Saval law:

(5) $$\vec{B_{e}}=\int_{L}\frac{u_{0}I}{4\pi }\frac{dl\times \vec{e_{r}}}{r^{2}},$$

Here, *I* represents the source current, *L* is the integration path, *dl* is the tiny line element of the source current, $\vec{e_{r}}$ is the unit vector of the current element pointing to the field point to be sought, and μ_0_ is the vacuum permeability with a value of 4π × 10^−7^ N-A^−2^. Using this, the current in each solenoid coil can be computed for the attitude control of the driving magnetic sphere by prescribing the moments and forces of the motion beforehand through inverse matrix operations:

(6) $$\zeta =A_{T,F}^{-1}\left[\begin{array}{c} T\\ F_{m} \end{array}\right],$$

In Equation (6), **ζ** denotes the current matrix of n coils in A; T is the magnetic moment matrix applied to the microrobot in N/m; and F_m_ is the magnetic force matrix applied to the microrobot in N. The detailed derivation of Equation (6) is presented in the auxiliary information theory formulation. By manipulating the position and attitude of the NdFeB magnetic sphere through Equations (4) and (6), it becomes possible to control the position and attitude of the magnetic microrobot using a more potent magnetic field.

#### 2.2.4. Force Analysis of Magnetic Microrobot in Electromagnetic Drive Systems

For a microrobot subjected to forces in a liquid environment as in Figure 3c, the combined force expression (7) is as follows:

(7) $$\vec{F_{m}}+\vec{F_{b}}+\vec{F_{d}}+\vec{G}=m\vec{a},$$

1. $\;F_{d}$ Liquid Viscosity

Assuming that the motion environment in which the microrobot operates is a viscous incompressible fluid, the fluid viscous force is calculated by Navier–Stokes equations:

(8) $$F_{d}=-\frac{1}{2}\rho_{f}{\left(v-v_{f}\right)}^{2}SC_{d},$$

In this equation, the velocity of motion v is in m/s; the fluid flow velocity *v_f_* is in m/s; the fluid density is in kg/m^3^; the front-end area S is in m^2^; and the drag coefficient is C_d_, where the drag coefficient C_d_ equation is given in the auxiliary information theory equation.

2. F_m_ magnetic drive

Assuming the microrobot function as a magnetized body, its magnetic strength is characterized by the magnetic moment $\vec{M}=\left[\begin{array}{c} m_{x}\\ m_{y}\\ m_{z} \end{array}\right]$, measured in units of A·m^2^. In this scenario, the microrobot is considered to consist of an ideal hard magnetic material with a fixed magnetic moment. The external magnetic field acting on the microrobot’s magnetized body is expressed as follows:

(9) $$T=M\times B_{q}=Sk\left(M\right)B_{q},$$

Here, *T* represents the magnetic moment applied to the microrobot in N-m, and *B_q_* is the magnetic induction strength of the magnetic sphere at the microrobot’s position in Tesla (T). Since the magnetic induction strength of the solenoid coil is negligible compared to the magnetic sphere, the vector cross-product is replaced with an antisymmetric matrix:

(10) $$Sk\left(M\right)=Sk\left(\left[\begin{array}{c} m_{x}\\ m_{y}\\ m_{z} \end{array}\right]\right)=\left[\begin{array}{ccc} 0 & {-m}_{z} & m_{y}\\ m_{z} & 0 & {-m}_{x}\\ {-m}_{y} & m_{x} & 0 \end{array}\right],$$

In this equation, *x*, *y*, and *z* denote the directions of the basis in three-dimensional space. The force *F*_m_ acting on the magnetic moment is measured in Newton (N):

(11) $$F_{m}=\left(M\cdot \nabla \right)B_{q},$$

The microrobot lacks an internal current, indicating no internal field source, and adheres to the flux continuity theorem as per Maxwell’s equations ∇∙*B* = 0 and the Hamiltonian operator algorithm:

(12) $$F_{m}={\left[\begin{array}{ccc} \frac{\partial B_{q}}{\partial x} & \frac{\partial B_{q}}{\partial y} & \frac{\partial B_{q}}{\partial z} \end{array}\right]}^{T}M$$

The magnetic moment M of the microrobot and the magnetic flux density Bq generated by the permanent magnet sphere at the position of the microrobot can be obtained through measurements. By substituting these values into Equations (9) and (12), the magnetic torque and force acting on the microrobot under the influence of the permanent magnet sphere can be calculated. Since the magnetic field intensity generated by the electromagnetic coil is much smaller compared to the permanent magnet sphere, the influence of the electromagnetic coil on the microrobot is not considered here.

3. G is gravity and F_b_ is buoyancy and possibly friction

Considering the microrobot’s diminutive size, its own weight can be counteracted by magnetic fields and buoyancy to minimize or even neglect the friction it encounters against the container walls. Here, we do not account for friction.

## 3. Results

### 3.1. Microrobot Attitude Control

**Figure 4:**
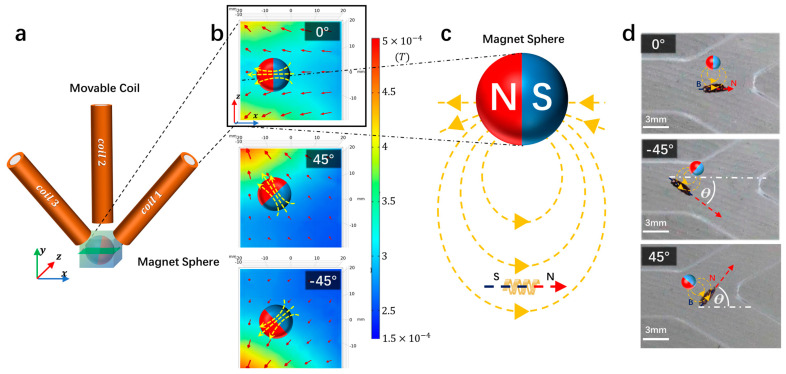
(**a**) Microrobot driving mechanism. (**a**) Three-axis electromagnetic coils are energized to generate a magnetic field for driving the magnetic sphere in electromagnetic simulation. (**b**) Schematic diagram of the relationship between magnetic field gradient and magnetic sphere direction (0°, 45°, −45°), with three different directions of magnetic field gradient controlling the rotation direction of the magnetic sphere. (**c**) The magnetization direction of the microrobot is along the axial direction. Under the influence of the external magnetic field of the magnetic sphere, the axial orientation of the microrobot will align with the direction of the external magnetic field of the magnetic sphere. (**d**) Under the influence of the magnetic field of the magnetic sphere, the microrobot will always maintain an orientation (0°, −45°, 45°), where the magnetization direction is consistent with the direction of the external magnetic field of the magnetic sphere (scale bar: 3 mm).

The magnetic field generated by the triple coils can be simulated by finite elements according to Equation (9). In the simulation, the magnetic field of the coils in a 40 mm square area centered on the NdFeB magnetic sphere is studied (Figure 4a). The direction of the magnetic field in the center area can be obtained by changing the current magnitude of the three coils. The magnetization direction of the NdFeB magnetic sphere will be rotated in the same direction towards the direction of the external magnetic field (e.g., Figure 4b).

Since the magnetization direction of the microrobot is along its long axis, the rotation of the NdFeB magnetic sphere will cause the long axis of the microrobot to follow the direction of the external magnetic field of the NdFeB magnetic sphere. By changing the current magnitude of the three coils, the direction of the NdFeB magnetic sphere is controlled to sequentially point to three intersections (0°, 45°, −45°). The effectiveness of this simulation was also verified through experiments. The experimental results show that the direction of the microrobot always remains opposite to the north pole of the magnetic sphere, pointing once to (0°, −45°, 45°) (Figure 2d). Based on this relationship, during the movement of the microrobot, the direction of the microrobot can be precisely controlled by varying the current in the electromagnetic coils. However, as the NdFeB magnetic sphere rotates, slight changes occur in the strong magnetic field points below the NdFeB magnetic sphere, resulting in a slight error in the position of the microrobot, with a maximum error of about 5 mm.

### 3.2. Microrobot 2D Targeting Experiments

The electromagnetic drive system utilized in this study effectively positions the microrobot during its motion, adjusting its orientation in response to changes in the track direction. To verify the control accuracy of the system and to better control the microrobot, the research team conducted preliminary experiments. The specific method is as follows: First, simulations were performed on individual electromagnetic coils to determine the magnitude of the current inside the coils (Supporting Information Figure S2), with a peak current of 10 A. Next, simplified movement experiments were conducted in a two-dimensional track (as shown in Figure 3d). In the electromagnetic drive system designed in this study, three stepper motors are connected to toothed belts. The toothed belts are also connected to the parallel arms with added electromagnetic coils. As the stepper motors move, the position of the permanent magnet sphere at the end of the delta structure changes. The position of the microrobot also changes accordingly. The principle of microrobot driving control is as follows: Firstly, place the two-dimensional track with the microrobot installed in the sample holder, aligning the center position of the track with the center of the delta. Secondly, the stepper motors rotate according to the motion distance calculated by the control program. The three electromagnetic coils with magnetic spheres slowly approach the microrobot under the drive of the toothed belt. Obtain the position coordinates of the microrobot through camera observation. And pre-plan the movement path of the microrobot according to the track information. Finally, input the obtained path nodes into the control program, and the drive system will move sequentially between each pair of points. The end magnetic sphere will pull the microrobot to move from point 1 to point 2, then from point 2 to point 3, and so on, until it reaches the endpoint. When inputting the coordinates of the trajectory nodes, the current in each electromagnetic coil can also be controlled according to the direction of each segment of the trajectory to ensure that the direction of the end magnetic field always remains consistent with the forward direction of the microrobot. Such a control strategy can ensure that the attitude of the microrobot always faces the forward direction. It allows the microrobot to pass through some narrow obstacles. Based on these experimental results, we conducted experiments on the movement of the microrobot in a two-dimensional track.

**Figure 5:**
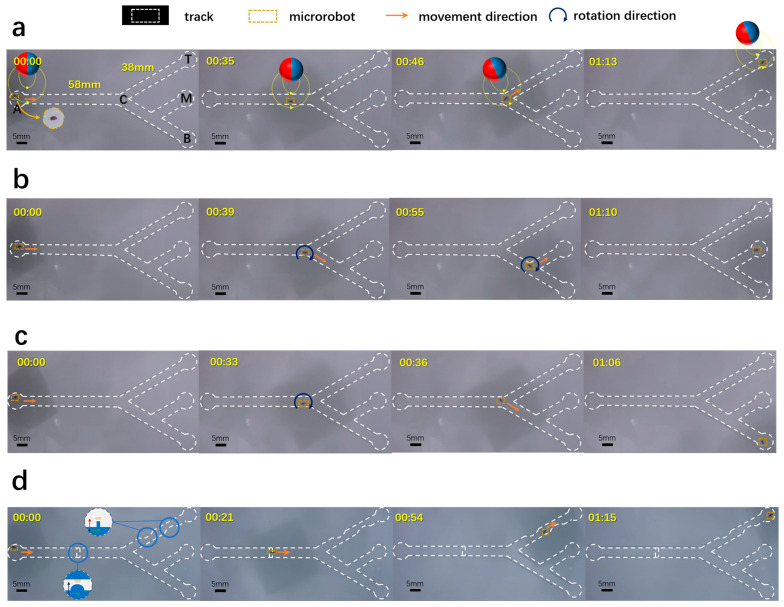
Experimental track of the electromagnetic drive system. The magnetic control system guides the microrobot to three predetermined localization points during movement control. Throughout this process, the motion of the magnetic sphere propels the microrobot, and the rotation of the magnetic sphere governs the microrobot’s orientation. Panel descriptions are as follows: (**a**) The microrobot transitions to point T. (**b**) The microrobot maneuvers to point M. (**c**) The microrobot advances to point B. (**d**) Leveraging control over the microrobot’s orientation, it successfully navigates through a height obstacle (with an obstruction height of 1 mm) and a narrow area (with the narrowest point measuring 3.5 mm, scale bar: 5 mm).

The microrobot achieves a motion speed of approximately 1.3 mm/s. The track depicted in Figure 5 is constructed from epoxy resin, featuring a main pipeline cross-section, which is a semicircular notch with a diameter of 5 mm, and the branch pipeline cross-section with a 6 mm diameter semicircular notch and a 5 mm diameter semicircular notch. This design approximates the human hepatic vein. The microrobot is placed in the track in advance, distilled water serves as the movement environment, and the branch routes exhibit a 60° angle. In Figure 5a, the microrobot reaches the top point (T) with a single turn. In Figure 5b, the microrobot reaches the middle point (M) after two steering maneuvers, ensuring that its long axis aligns with the track direction during each segment. In Figure 5c, the bottom point (B) is reached through a single steering action.

In anticipation of the microrobot’s future operational environments, which may not exclusively replicate the ideal conditions of a healthy blood vessel, we acknowledge the frequent occurrence of vessel constrictions for various reasons. Failure to achieve control over the microrobot’s orientation under such circumstances would curtail its applicability. In Figure 5d, we introduce two types of motion obstacles into the original experimental track. The first obstacle is a height restriction on the main pipe, with a defined obstruction height of 1 mm. The second obstacle is a lateral impediment on the branch pipe, strategically positioned at the narrowest point, measuring 3.5 mm. During the experiment, the microrobot adeptly navigated both obstacles, successfully reaching the targeted point T at a consistent speed of 1.3 mm/s throughout the entire process.

### 3.3. Microrobot Three-Dimensional Targeting Experiments

**Figure 6:**
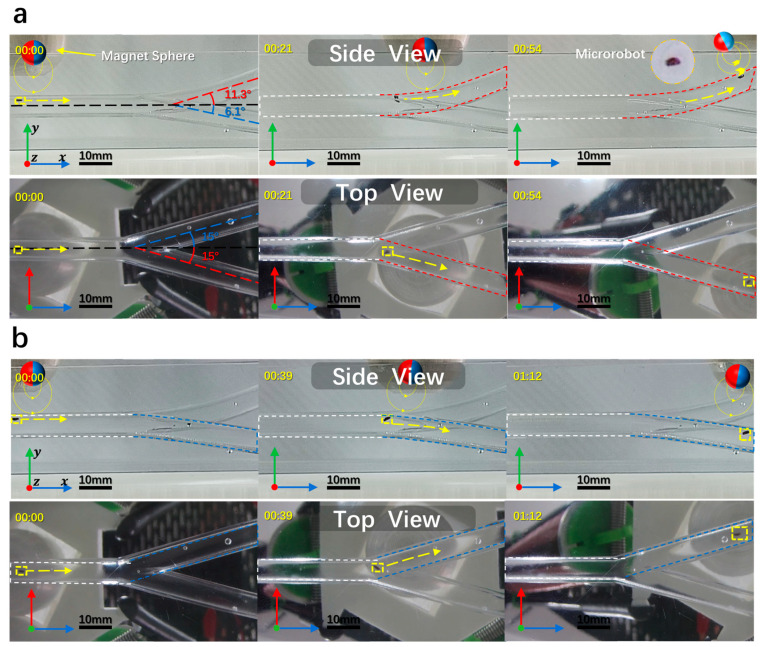
Electromagnetic three-dimensional drive system. Yellow dotted boxes are the positions of microrobot. Yellow dotted arrows are the movement directions of microrobot. (**a**) Side-view angle and elevation angle for observing the upward targeting motion of the microrobot. (**b**) Side-view angle and elevation angle for observing the downward targeting motion of the microrobot. (scale bar: 10 mm).

To validate the microrobot’s driving capabilities, we designed a three-dimensional (3D) pipeline to conduct experiments. The dimensions were 6 mm and the branch angles are shown in Figure 6. Crafted from epoxy resin, the track presented challenges during experimentation. The microrobot, driven by the electromagnetic drive system, reached the upper and lower target points at an average speed of 1.3 mm/s. Unexpected issues emerged during the experiment, notably collisions with the upper tube wall, impeding microrobot movement and complicating attitude control. Experiment findings indicated that maintaining a distance of approximately 10 mm between the NdFeB magnetic sphere and the microrobot allowed for relatively stable movement in the 3D pipeline. However, at a distance exceeding 10 mm, the microrobot struggled to be pulled due to the extended gap, leading to uncontrolled movement. Conversely, when the distance was less than 10 mm, the NdFeB magnetic sphere’s traction force was excessive, causing the microrobot to adhere to the inner pipe surface, increasing friction, hindering effective movement, and potentially causing damage. Two solutions are envisioned: using electromagnetic coils for more sensitive microrobot control, which requires a higher-power external supply and coils with more turns, or enhancing the motion accuracy of the motors driving the electromagnetic coils to a millimeter-level error. The latter solution necessitates pre-planning the motion trajectory, real-time acquisition of motion information, and feedback-adjusted pre-motion trajectories to enhance movement precision.

## 4. Discussion and Conclusions

In this study, the driving experiments are confined to a static water medium, neglecting the complexity of the actual biological environment characterized by factors like flow rate and viscosity. In numerous research experiments, only motion studies are conducted in stagnant water, primarily due to size-related constraints. Size effects mainly manifest in various aspects. These include viscosity, flow velocity, and eddy currents, among others, all of which impact the mobility of microrobots [28,29]. Consequently, future research will incorporate fluid dynamics simulation and experimentation to elucidate the motion characteristics across various scales. Moreover, the materials utilized in microrobots are influenced by size effects. The body materials of microrobots primarily comprise non-metallic substances like photosensitive resin and polymers. In drug-targeting applications, biodegradable materials like Poly (lactic-co-glycolic acid) (PLGA), Polyethylene glycol (PEG), and Chitosan are frequently employed [30]. This study solely employs a direct current power supply, limiting the microrobot to basic steering maneuvers through current variations and precluding more intricate movements. To address this constraint, employing an alternating current power supply and integrating artificial intelligence algorithms like deep learning in future research is recommended. This would enhance the precise recognition and positioning of microrobots, enable movement trajectory prediction in tissue environments, facilitate accurate current calculation, and allow for more precise and flexible microrobot control. The cameras employed in the experiments are fixed along two directions of the drive system to capture and monitor the real-time position of the microrobot. Future research could explore the design of mechanical arms or the installation of movable cameras at the endpoints of triangular structures to broaden the observation range. While the NdFeB magnetic sphere offers an efficient means of controlling the microrobot due to its strong magnetism, the magnetic field gradient surrounding permanent magnets fluctuates significantly with distance, rendering them vulnerable to disturbances caused by changes in distance. Future research could explore the enhancement of stepper motors and gear structures to enhance movement precision alongside intelligent recognition. Real-time monitoring of the microrobot’s positions and providing feedback to the control system can mitigate control system errors. The experimental pipeline dimensions utilized in this study closely resemble those of human hepatic veins. Subsequent endeavors will concentrate on resolving the aforementioned issues to enhance and refine the drive system’s design scheme.

In conclusion, this system indirectly controls the position and attitude of a magnetic microrobot through structured positional movements, relying on real-time enhancement of the magnetic field by electromagnetic coils to control the direction of the NdFeB magnetic sphere, making the drive mechanism more straightforward and effective. By utilizing the combination of electromagnetic coils and a permanent magnet sphere, the research team achieved synchronized control of the microrobot’s movement position and attitude direction. The entire drive system, consisting of two components, utilizes a stepper motor to drive the delta structure on one hand, changing the position of the end-mounted permanent magnet to induce microrobot movement. On the other hand, a programmable DC power supply is used to control the current strength in the electromagnetic coil, changing the direction of the magnetic field at the end, thereby driving the change in attitude of the permanent magnet, and consequently, the attitude of the microrobot. E-dent 400 photosensitive resin blended with NdFeB particles (50%:50% wt) was used in this study, representing a novel mixed photopolymer solution. A microrobot was fabricated using a single-layer 4D printing method that utilizes focused light, and experiments were conducted in 2D and 3D tracks, simulating the morphology of the human liver veins. The average speed of the microrobot during movement was 1.3 mm/s (movement error ± 0.5 mm). Experimental validation confirmed the drive system’s ability to achieve relatively accurate control over the microrobot’s movement position and attitude rotation. Verified results demonstrate that the designed novel drive system is suitable for driving robots of millimeter-level and smaller sizes. The experimental results emphasize that the novel drive system has achieved higher precision in controlling the microrobot’s movement position and attitude rotation.

These findings provide valuable insights for the design of future electromagnetic drive systems and the application of microrobots in the medical field, paving the way for further innovation and breakthroughs in the intervention of the microrobotics field.

## Data Availability

All data for this study have been experimentally generated and have been included in this paper.

## Supplementary Materials

The following supporting information can be downloaded at https://www.mdpi.com/article/10.3390/mi15040438/s1.
