# Supplementary material for: Control of Self-Winding Microrobot Using an Electromagnetic Drive System: Integration of Movable Electromagnetic Coil and Permanent Magnet
Source: Micromachines (Basel). 2024 Mar 25;15(4):438. doi: 10.3390/mi15040438 (PMC11052315; doi:10.3390/mi15040438)
Supplement: Supplementary file 1 [file micromachines-15-00438-s001.zip › SI .pdf]

Supporting Information

Control of a Self-winding Microrobot Using an Electromagnetic Drive System: Integration of a Movable Electromagnetic Coil and Permanent Magnet.

Parameters	Numerical value	Unit (of measure)
Coil Diameter	40	mm
Coil length	200	mm
peak current	5	A
Number of turns	350	

Table S1. Parameters of solenoid coils

1. Electromagnetic drive principle

The magnetic field $\overrightarrow{\boldsymbol{B_e}}$ produced by a single electromagnetic coil at any point in space can be calculated using the Biot-Saval law:

$$\overrightarrow{\boldsymbol{B_e}} = \int_L \frac{u_0 I}{4\pi} \frac{dl \times \overrightarrow{e_r}}{r^2} \tag{1}$$

where I is the source current, L is the integration path, dl is the tiny line element of the source current, $\overrightarrow{e_r}$ is the unit vector of the current element pointing to the field point to be solved, and μ_0 is the vacuum permeability with a value of $4\pi \times 10^{-7}$ N-A^{-2}.

In equation (1), the magnitude of the magnetic induction is proportional to the current in the electromagnetic coil I, it can be expressed as the magnetic induction produced per unit of current($\widetilde{\boldsymbol{B_e}} \approx$T/A) and the product of the current (I_e in A):

$$\boldsymbol{B_e} = \widetilde{\boldsymbol{B_e}} i_e \tag{2}$$

Assuming the core of the electromagnetic coil is an ideal soft magnetic material with a linear variation range, the magnetic field at any point in the entire motion space can be obtained through the superposition of multiple electromagnetic coils:

$$\boldsymbol{B} = \sum_{e=1}^{n} \boldsymbol{B_e} = \sum_{e=1}^{n} \widetilde{\boldsymbol{B_e}} i_e \tag{3}$$

The core material in this study is manganese-zinc ferrite (NiZn(MgZn)) with a permeability of 800 H/m. For Eq. (3), a linear summation representation is obtained:

$$\boldsymbol{B} = [\widetilde{\boldsymbol{B_1}} \quad \cdots \quad \widetilde{\boldsymbol{B_e}}] \begin{bmatrix} i_1 \\ \vdots \\ i_n \end{bmatrix} = \boldsymbol{\beta\zeta} \tag{4}$$

In Eq. (4), $\boldsymbol{\beta}$ denotes the matrix of magnetic induction strengths produced by unit currents of n coils, and ζ denotes the matrix of currents of n coils. The superimposed magnetic field of multiple electromagnetic coils at any point in space can be calculated according to (4). Similarly, the derivative in a given direction can be expressed as a contribution from each current, for

example in the x direction:

$$\frac{\partial \boldsymbol{B}}{\partial x} = \begin{bmatrix} \frac{\partial \widetilde{\boldsymbol{B}}_1}{\partial x} & \cdots & \frac{\partial \widetilde{\boldsymbol{B}}_e}{\partial x} \end{bmatrix} \begin{bmatrix} i_1 \\ \vdots \\ i_n \end{bmatrix} = \boldsymbol{\beta}_x \boldsymbol{\zeta} \tag{5}$$

If we consider the magnetic strength of the magnetic sphere itself as $\overrightarrow{M_S}$, then the magnetic torque and magnetic force of the magnetic sphere can be found as follows:

$$\begin{bmatrix} \boldsymbol{T} \\ \boldsymbol{F_m} \end{bmatrix} = \begin{bmatrix} Sk(\overrightarrow{\boldsymbol{M_S}})\boldsymbol{\beta} \\ \boldsymbol{M}^T \boldsymbol{\beta_X} \\ \boldsymbol{M}^T \boldsymbol{\beta_Y} \\ \boldsymbol{M}^T \boldsymbol{\beta_Z} \end{bmatrix} \begin{bmatrix} i_1 \\ \vdots \\ i_n \end{bmatrix} = \boldsymbol{A_{T,F}} \boldsymbol{\zeta} \tag{6}$$

This implies that, for the attitude control of the driving magnetic sphere, the moment and force of the motion can be pre-specified through inverse matrix operations, and the current of each electromagnetic coil can be calculated:

$$\boldsymbol{\zeta} = \boldsymbol{A}_{T,F}^{-1} \begin{bmatrix} \boldsymbol{T} \\ \boldsymbol{F_m} \end{bmatrix} \tag{7}$$

2. Liquid viscosity

Equation (8) in the main text addresses the drag coefficient C_d in fluid motion. The formula is expressed as:

$$C_d = \frac{24}{R_e} + \frac{6}{1 + \sqrt{R_e}} + 0.4 \tag{8}$$

In this equation R_e is the Reynolds number:

$$R_e = \frac{\rho_f (v - v_f) L}{u_f} \tag{9}$$

where μ_f is the hydrodynamic viscosity coefficient and L denotes the characteristic length of the microrobot.

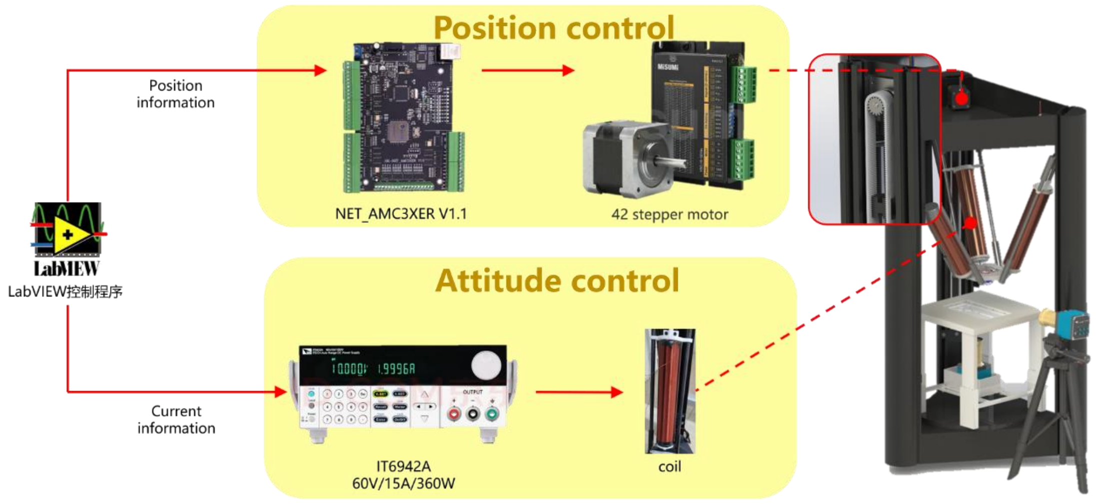

Fig. S1. Modules of the electromagnetic drive system components

3. Video files

Movie S1.

Simulation analysis of single-layer optical focusing microrobot curling using finite element software compared with actual curling.

Movie S2.

Targeted motion of a microrobot reaching different targeting points in a two-dimensional region.

Movie S3

Targeted motion of a microrobot through a two-dimensional region with height and pipe obstruction.

Movie S4

Targeted motion of a microrobot reaching different targeting points in a three-dimensional pipeline.

Movie S5

Finite element simulation analysis of magnetic field for the moving process of electromagnetic drive system.

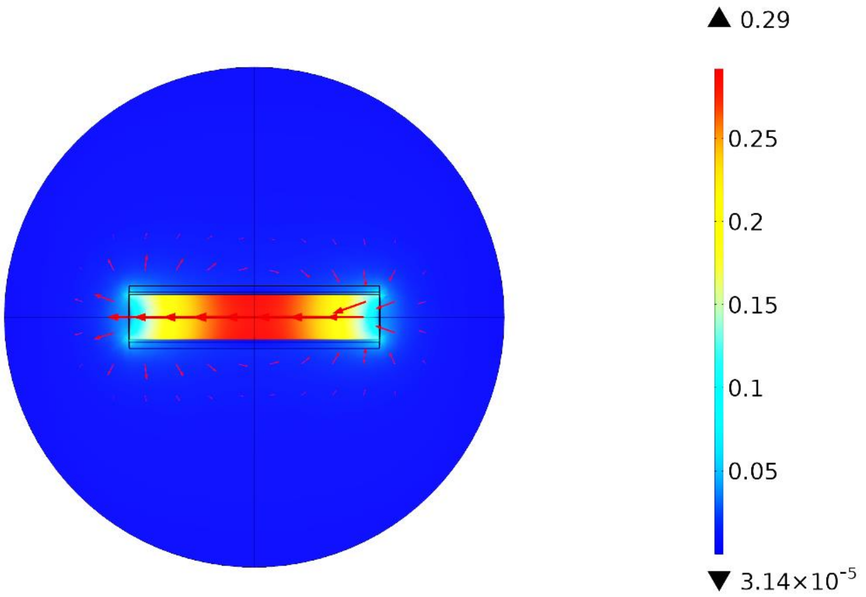

Fig. S2. The magnetic field generated when a single electromagnetic coil is energized with a current of 10 A.
