# Supplementary figures and images for: Control of Self-Winding Microrobot Using an Electromagnetic Drive System: Integration of Movable Electromagnetic Coil and Permanent Magnet
Source: Micromachines (Basel). 2024 Mar 25;15(4):438. doi: 10.3390/mi15040438 (PMC11052315; doi:10.3390/mi15040438)

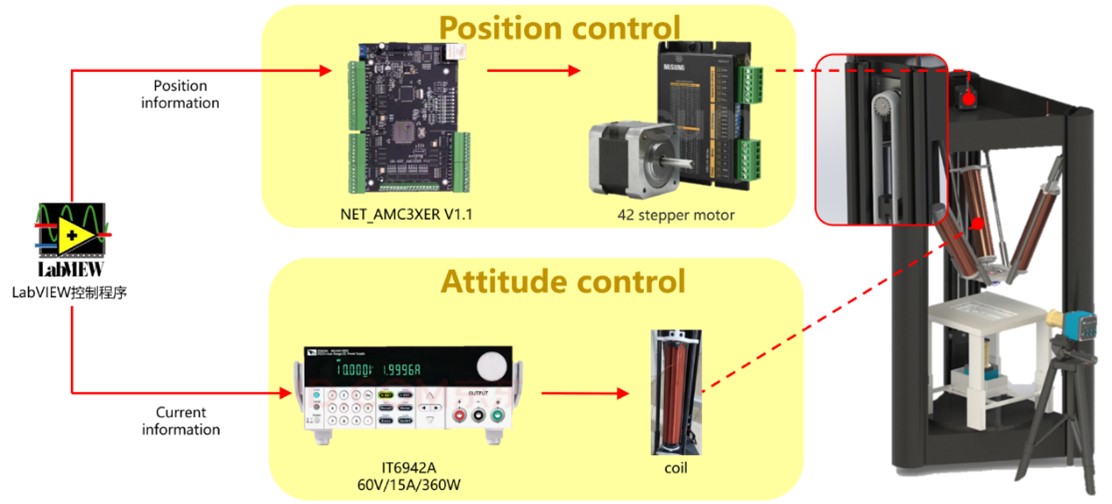

Supplement: Supplementary file 1 [file micromachines-15-00438-s001.zip › SI 01.jpg]

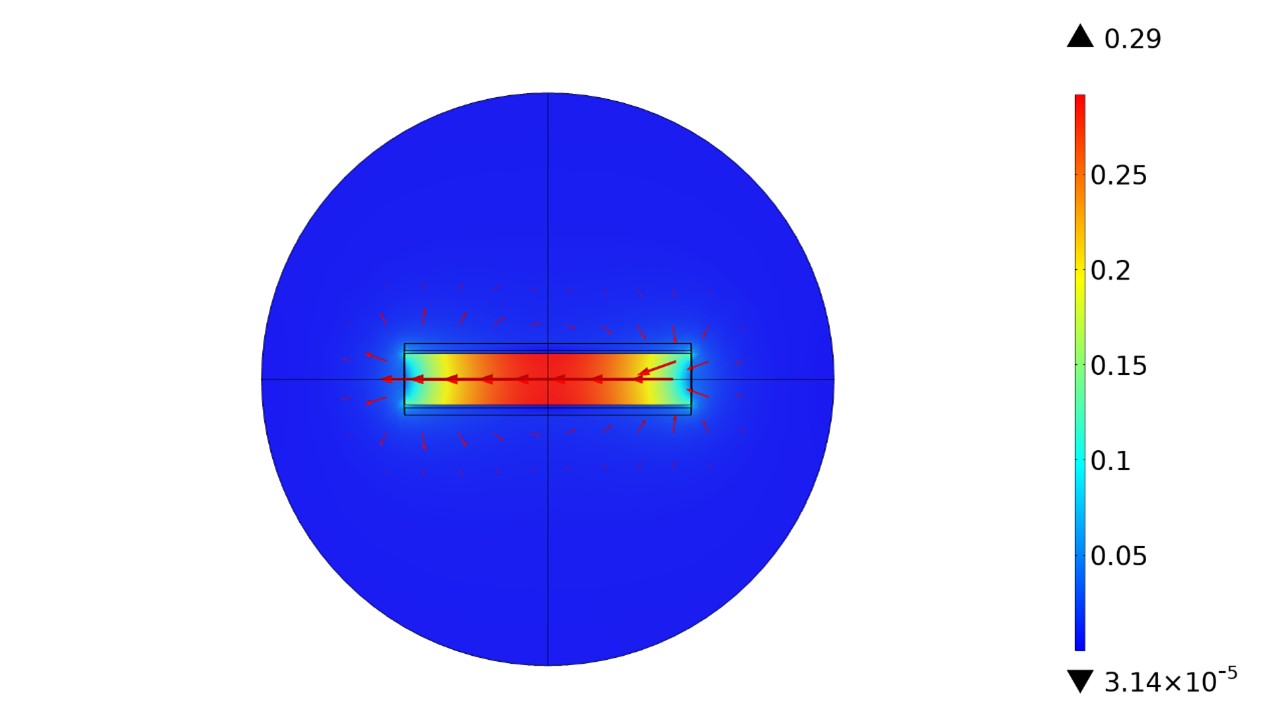

Supplement: Supplementary file 1 [file micromachines-15-00438-s001.zip › SI 02.jpg]
